# Prevalence of stress in Casablanca medical students: a cross-sectional study

**DOI:** 10.11604/pamj.2014.19.149.4010

**Published:** 2014-10-15

**Authors:** Dalal Ben Loubir, Zeineb Serhier, Samir Diouny, Omar Battas, Mohamed Agoub, Mohammed Bennani Othmani

**Affiliations:** 1 Medical Informatics Laboratory, Clinical Neuroscience and Mental Health Laboratory, Casablanca Medical Faculty, Hassan II University, Morocco; 2 Chouaib Doukkali University, Faculty of Letters and Human Sciences, Department of English, El Jadida, Morocco; 3 Clinical Neuroscience and Mental Health Laboratory, Casablanca Medical Faculty, Hassan II University, Morocco

**Keywords:** Stress, academic skills, medical students, test anxiety, Morocco

## Abstract

**Introduction:**

Recently, an important literature data has reported that medical students experience stress more than students in other disciplines. In contrast, there is a significant shortage of the stress impact on the academic performance. The primary purpose of our study was to determine the prevalence of stress among Casablanca Medical students and to investigate if there is an association between stress and academic skills.

**Methods:**

A total of 275 participants studying at Casablanca Medical School were included. The study was conducted using a self-administered, anonymous questionnaire, which included four subscales on academic skills perception (Academic competence, Test competence, Time management and Strategic study habits) and a Test Anxiety scale to assess the degree of stress related to exams among medical students.

**Results:**

The overall findings showed that 52.7% of respondents were stressed by examinations, and the highest stress prevalence was among the fifth-year medical students. Measures of comparative stress degrees between male and female students did not show any statistical significant differences (p=0.34). Correlation analysis revealed negative association between stress and academic competence (-0.394), test competence (-0.426), time management (-0.240), strategic study (-0.183) respectively (p<0.001).

**Conclusion:**

Medical educators and psychologists have to increase clinical awareness of stress among medical students, by establishing strategies for stress management.

## Introduction

In recent years, stress has been studied from different perspectives (psychiatry, neurology, psychology etc.). Stress occurs when pressure exceeds one´s perceived ability to cope with daily demands experienced by the individual [1]. Stress has both emotional and physical symptoms such as fatigue and anxiety-related disorders. Studies measuring stress experienced by medical students and dental students have been frequently reported in the academic health literature. Specifically, these studies reported that medical students face a serious academic challenges that render them more vulnerable to stress and anxiety than students of other disciplines [2, 3]. Moreover, different studies support an inverse association between anxiety and performance [4, 5]. They further indicated that medical students experienced increased symptoms of depression and anxiety [6–8], associated with high levels of stress [9–11], which can lead to disruptions in both physical and mental health and may diminish the student's sense of worth, thus affecting his/her academic achievement [12].

Clinical training might be a source of high stress level in medical students [13]. During this period, medical students assume responsibility and consequently have to cope with the pressure of work, lack of leisure time and work relationships [14]. Other studies, however, argued that the potential stressors among medical students can be related to academic pressures [15, 16], poor study habits, unsuitable teaching methods, unsatisfactory study environment and overloaded academic curriculum which are also reported to cause exam anxiety in medical students [17]. Stressors may also include doctor-patient miscommunication and the acquisition of applied clinical skills [18]. Likewise, the lack of time was cited as the main factor reducing quality of life among medical students [19]. All these factors may have an adverse effect on both academic performances and medical training of medical students, especially exams pressure and overloaded academic curriculum.

Since the curriculum puts too much emphasis on rote learning, and not enough on interaction and logical reasoning, some students experience difficult remembering facts. Furthermore, language problems may be another source of stress. In Morocco, Modern Standard Arabic is the language of instruction for scientific subjects in schools, while at the medical school; French is a mean of instruction. At Moroccan medical schools, students pursue their studies during two semesters each year, and have to take two exams, one in January and another in May. Furthermore, when student gets a score lower than 10/20 in a particular subject, in the first or in the second semester should sit for make-up exam during the exams period at the end of the academic year. Very few studies, involving medical student distress, were undertaken in the Arab countries which are different from developed countries. The aim of the present study was to measure the prevalence of stress related to exams among medical students and to assess its relationship with student's academic skills.

## Methods

We conducted a cross-sectional survey during the second semester of the academic year 2012-2013, in January. Students from the first (1^st^) to the fifth (5^th^) years studying at Casablanca medical school were recruited for this study. One thousand eight hundred forty five students were enrolled in the 1^st^, 2^nd^, 3^rd^, 4^th^ and 5^th^ years. A stratified cluster sampling technique was used to select students’ sample from each academic year; the groups were randomly chosen. Students from the sample belonged to six tutorial groups in the 1st and in the 2^nd^ year, and to three hospital units in each 3^rd^, 4^th^ and 5^th^ year students. An anonymous self-administered questionnaire containing 30 items was administered to participants of selected groups. All participants were informed about the aim of the study and were assured about anonymity and confidentiality, and provided a verbal consent before their participation to the study. Regarding ethical permission, the study was approved by

### « Comité d’éthique pour la recherche biomédicale Casablanca »

The Test anxiety scale was adapted from a previously validated test-anxiety inventory [20]. Test anxiety scale was used in previous works [17, 21], and was employed in this study to measure stress related to exams. The Test anxiety is a set of 10 items, like worry, nervousness, depression, etc... We carried out scales those represent academic skills: Academic Competence, Test Competence, Time Management and Strategic Study habits, which were adapted from previous works [21, 22]. These scales have been previously correlated with Test anxiety scale [21]: - academic competence: contains 5 items that are related to the ability of the student to manage and understand the course taught; - test competence: with 4 items related to the effort made by the student to understand the course content and to prepare for exams; -time management: in 5 items. The items concern time organization between study and leisure time, and time management for the exams preparation; - strategic study: with 5 items that concern methods and tools used by students to plan the exams preparation; - all of the 19 items were measured using a 5-point Likert scale from 1 (strongly agree) to 5 (strongly disagree).

Since there is no validity version for the French version of the used scales, these instruments were translated into French according to Beaton et al. [23]. The survey instrument also included questions, whose purpose was to obtain information on age, gender and year of enrollment. For Test anxiety scale, each item response could range from 1 (Not at all typical of me) to 5 (Very much typical of me). Test anxiety score was calculated as the mean of the items responses scores. A student with a test anxiety score higher than 3 (3/5 middle of Likert scale), is considered stressed. Academic skills, which include academic competence, test competence, time management and strategic study habits, were calculated as the mean of the corresponding items responses scores. Some of the academic skills items were reversed to indicate that higher scores correspond to better performance. Reliability analysis for scales was carried out by calculating Cronbach´s?, a value of 0.70 or greater was considered adequate [24]. Pearson Correlation was used to evaluate associations between variables. Data analysis was carried out by STATA (version 11.0).

## Results

A total of 275 participants completed the survey instrument (participation rate was 98%). Demographic characteristics by year of enrollment are summed up in Table 1. The mean age of respondents was 20.5 years (±1.9 years) (M±SD), 69.5% of the students were females. The Cronbach's α for assessing the internal consistency reliability were 0.76, 0.69, 0.69, 0.61 and 0.56 for test anxiety scale, academic competence, test competence, time management and study habits respectively. The mean score for test anxiety was 3.05 (±0.54) (Table 2); 52.7% of respondents were stressed (score>3) by examinations, 44.0% of males and 56.5% of females (p=0.34). Table 2 revealed that more than three quarters of respondents (81.1%) felt worry by examination. Furthermore, the majority of respondents (74.9%) reported experiencing anxiety during examinations even though they thought they were well-prepared and 73.7% were afraid to fail the exam.

**Table 1:** Descriptive statistics by year of enrollment

Variable		1^st^-Year	2^nd^-Year	3^rd^-Year	4^th^ -Year	5^th^-Year	Overall
	N	59	67	49	49	51	275
Age	M (SD)	17.9 (0.9)	19.8 (1.0)	21.0 (0.9)	22.0 (0.7)	22.8 (1.0)	20.5 (1.9)
Gender(F)	N (%)	37 (63.8)	53 (79.1)	32(65.3)	33 (67.3)	35 (68.6)	191 (69.5)

N: Number of students who answered that question; M: Mean; SD: Standard Deviation; F: Female

**Table 2:** Medical students’ perceptions to determine test anxiety

			*Response*	*Distribution*		
Item	1 Not at all typical of me (%)	2 Not very typical of me (%)	3 Some- what typical of me (%)	4 Fairly typical of me (%)	5 Very much typical of me (%)	Mean (SD)
Afraid to fail exams	10.6	15.7	27.0	22.6	24.1	3.3 (0.4)
Nervousness	10.5	18.9	20.0	22.9	27.6	3.4 (0.5)
Perspiration	31.8	32.5	14.6	9.1	12.0	2.4 (0.1)
Task-irrelevant cognitions	34.2	19.6	21.1	11.6	13.5	2.5 (0.1)
Panicky	12.0	14.2	25.5	20.0	28.4	3.4 (0.5)
Upset Stomach	41.4	17.9	15.8	13.2	11.7	2.4 (0.1)
Increased heartbeats	17.9	16.8	19.3	19.3	26.6	3.2 (0.4)
Depression	32.4	23.9	18.4	11.0	14.3	2.5 (0.1)
Worry	9.5	9.5	14.2	18.5	48.4	3.9 (0.9)
Anxious even when well-prepared	11.3	13.8	18.5	22.2	34.2	3.5 (0.6)

Mean test anxiety = 3.0 (0.5); Cronbach Coefficient Alpha = 0.7

The mean academic competence score was 3.2 (± 0.5) (Table 3), which indicated that only few students were comfortable with course content. Around 36.8% of the respondents claimed they were able to manage their course load, about 41% of students enjoyed their courses and 82.1% made an effort to understand the course material. The mean score for the test competence was 2.4 (± 0.1), indicating that the students had difficulties to prepare for examinations. More than half of the respondents (52.9%) had difficulty in managing the amount of study material.

**Table 3:** Academic competence, test competence, time management and strategic study

			*Response*	*Distribution*		
Item (Cronbach Coefficient Alpha)	1 Strongly Agree (%)	2 Agree (%)	3 Neutral (%)	4 Disagree (%)	5 Strongly disagree (%)	Mean
Managing course load	10.2	26.6	26.3	23.0	13.9	2.9 (0.3)
Comprehension	5.9	26.7	30.8	23.4	13.2	2.8 (0.4)
Interest	18.3	37.7	24.2	10.6	9.2	3.4 (0.6)
Enjoyment	13.2	27.9	24.6	20.6	13.6	3.0 (0.3)
Efforts	35.2	46.9	9.9	3.7	4.4	4.0 (0.9)
*Academic competence (0.69)*						*3.2 (0.5)*
Easily manage study material	7.7	14.2	22.3	34.7	21.2	2.5 (0.2)
Test preparation	2.2	11.7	21.2	42.7	22.3	2.3 (0.3)
Coping with examination preparation	3.6	12.4	22.6	39.4	21.9	2.3 (0.3)
Difficulty in managing study material	25.9	27.0	27.4	10.9	8.8	2.5 (0.2)
*Test competence (0.69)*						*2.4 (0.1)*
Difficulty in combining study and leisure time	39.9	34.7	9.6	9.2	6.6	2.0 (0.2)
Studying regularly	32.1	43.4	13.9	6.6	4.0	2.0 (0.2)
Cramming for examinations	25.5	42.3	20.4	8.4	3.3	2.2 (0.3)
Organization	6.9	9.1	20.8	37.2	25.9	2.3 (0.2)
Early test preparation	10.6	27.5	19.4	24.2	18.3	2.9 (0.3)
*Time management (0.61)*						*2.2 (0.4)*
Judgment of test questions	26.5	36.0	22.1	9.2	6.3	3.7 (0.6)
Advance planning	9.2	36.0	32.7	14.3	7.7	3.2 (0.5)
Review	9.6	24.6	27.2	22.4	16.2	2.9 (0.3)
Knowledge assessment	30.9	44.1	9.6	10.3	5.1	3.8 (0.8)
Summarize	19.5	30.9	22.1	15.4	12.1	3.3 (0.4)
*Strategic study (0.56)*						*3.4(0.4)*

The mean score for time management was 2.2 (± 0.4), indicating that most of the students were not able to manage their time properly. The majority of respondents (74.6%) had difficulty corresponding on a balance between studies and leisure time and could not study regularly. Some of the respondents (38.1%) reported preparing early for an examination. The mean score for the strategic studying was 3.4 (±0.4) indicating that most of the students used some techniques of study habits to achieve academic success. Almost half of the students (50.4%) reported that they summarized the course material, and 34.2% reviewed the course material with their classmates.

There was a significant difference in medical students’ response (Table 4) across the five years. The 5^th^ year students experienced a higher test anxiety score (3.4 ± 0.7) as opposed to 1^st^, 2^nd^, 3^rd^and 4^th^ year students. Stress prevalence's among 1^st^, 2^nd^ , 3^rd^ , 4^th^ and 5^th^ years were 30.5%, 59.7%, 53.1%, 49% and 72.5% respectively (p=10-3). There was a poor academic skills among 5th year students concerning academic competence (2.9 ± 0.8), test competence (2 ± 0.7) and time management (1.9 ± 0.7), compared to the previous years. On the other hand, 1^st^ year students showed the highest academic skills, and the lowest test anxiety compared to other years. For strategic study habits, no significant difference between students in the different years was reported. Concerning correlation, Test anxiety was negatively associated with academic competence (-0.394), test competence -0.426, time management -0.240 (p <10^-3^) and strategic studying -0.183 (p <10^-2^). This indicates that academic skills decrease with increasing exams stress among medical students.

**Table 4:** Mean scores and standard deviation of test anxiety, academic competence, test competence, time management and strategic study across the academic years

Variable	First- Year	Second-Year	Third-Year	Fourth-Year	Fifth-Year	P-value
	Mean (SD)	Mean (SD)	Mean (SD)	Mean (SD)	Mean (SD)	
Test anxiety	2.7 (0.7)	3.1 (0.8)	3.1 (0.8)	2.9 (0.6)	3.4 (0.7)	<10^-3^
Academic competence	3.6 (0.6)	3.2 (0.7)	3.3 (0.9)	3.3 (0.6)	2.9 (0.8)	<10^-3^
Test competence	3 (0.6)	2.2 (0.6)	2.5 (0.8)	2.3 (0.8)	2 (0.7)	<10^-3^
Time management	2.8 (0.6)	2.2 (0.6)	2.4 (0.7)	2.2 (0.6)	1.9 (0.7)	<10^-3^
Strategic study	3.5 (0.6)	3.3 (0.7)	3.2 (0.9)	3.4 (0.7)	3.3 (0.6)	0.1446

## Discussion

The current study aimed to measure the prevalence of stress related to examinations among medical students and to identify its association with student's academic skills. Results of the study revealed that half (52.7%) of the respondents were stressed by examination. With regard to the stress prevalence among the different academic years, the obtained results were statically different (p <10^-3^). Negative associations were found between test anxiety and academic competence (-0.394), test competence (-0.426), time management (-0.240) and strategic study habits (-0.183) (p < 0.01). Concerning the used scales for measuring the test anxiety and the academic skills, acceptable reliability was achieved and all Cronbach's alpha were nearby 0.7, unless for time management and strategic study.

The present study supports the results of many previous works, in which medical students experience a high level of stress was demonstrated [2, 3]. The obtained results from the students of the 5^th^year support the view mentioned in the literature [25, 26], that this period is particularly stressful. This high stress level may be due to the high amount of courses taught in the 5^th^ year compared to the previous years; adding to that, the responsibility that, in the 5^th^ year students take during their internship in medicine, which render them more vulnerable. These factors affect both their academic performance and clinical training. Some researchers have tried to explain the high stress level obtained among the 5^th^ year of medical studies; these students have to deal with pressure of work, curriculum demands, lack of leisure time, professional relationships and career choices [14]. On the other hand we note that the highest academic competence, test competence and time management scores were among the 1^st^ year students, and then decrease to reach the lowest scores among the 5^th^ year students. The poorest academic skills found among the 5^th^ year of medical students may be due to the students’ inability to manage clinical training and the large amount of courses information; which may be resulting in an increase of the stress degree. The two next experienced groups characterized with some high level of stress were students of the 2^nd^ and 3^rd^ year (Table 4).

In the literature, similar findings reported a high level of stress among the students of the 2^nd^ year [15]. This result may be due to the fact that these students are stressed by the overwhelming amount of information they have to learn, furthermore, the 1^st^ and the 2^nd^ academic years are forming the pre-clinical phase, in which students are able to repeat the academic years once only, otherwise they must drop out of medical school. Adding to that, 2^nd^ year students have to succeed at all subjects in order to pass to the 3^rd^ year (clinical phase), which is not the case of the other academic years, where students can make it to the next class though they don't succeed at one subject at the most. In regard to the high stress level experienced by 3^rd^ year students, students here may find it difficult to deal with the clinical training, or they are not yet familiar with this new method of learning. Concerning 4^th^ year students, they seem familiar to the hospital learning environment which explains their low stress degree.

The medical students of the 1^st^ year, who are in the start of their medical education, seem to experience less stress comparing to other medical academic years, knowing that, at the study period, these students have yet to experience the pressure of exams period in the end of the academic year, which may explain their low stress degree. However, it is important to note that in a previous work, it was demonstrated that students who passed had significantly lower stress, anxiety, and depression symptoms than those who failed the 1^st^ year final examination (p <0.05) [27]. Thus, in our case, we can consider a second explanation and say: because students in 1^st^ year do not have any experience of the medical school, they have not yet found the medical education stressful, comparing to the other academic years. Add to this, the absence of clinical training that allowed students to have enough time for preparing exams, resulting in high academic skills. Moreover, we note a negative correlation between academic skills and test anxiety (p < 0.01). Similar results, except for the test anxiety and strategic study correlation, were seen in pharmacy students [21]. With regard to the negative correlation results, we can say that probably students who are significantly stressed have lower self-perceived efficacy, and therefore indicate low academic competence, test competence etc. Or, maybe students who perceived lower academic skills tend to get stressed more during exams. Higher levels of exams stress were reported across the 2^nd^, 3^rd^ and the 5^th^ years of enrollment. The situation requires help and support to students. It has been demonstrated that stress-management programs play a positive role in reducing the psychological distress of medical students [28]. Therefore, we recommend academic partners and medical educators involved in Casablanca Medical School to consider published literature on stress-management programs, in order to guide and establish helpful stress-management strategies to enhance and improve students’ well-being and academic skills.

At the same, in order to reduce the impact of clinical training pressures, 5^th^ year students should benefit the most from assistance throughout their training. Lowering stress will also contribute to a better physician-patient relationship [29]. Researchers recognized that stressors may induce higher level of stress and anxiety [30], which may affect students well-being, and therefore affect the quality of care medical professionals should offer [31, 32]. Further research involving a large proportion of medical students should be carried in Moroccan medical schools as well as in other universities in the Arab countries to investigate further medical training factors that impact student stress and student's academic performance, and to check for cultural differences. However, in order to investigate the overall student well-being, future research may take in consideration farther sources of stress (relation with friends/class, family health problems…), that show a significant association with high stress level in medical students and that impact student academic performance [33].

## Conclusion

Results of the present study can help medical educators and psychologists to increase clinical awareness of stress among medical students with a view to enhancing their well-being, and identifying strategies to cope with stress related to medical school and foster better communication.
